# Prospects of Immunology Education and Research in Developing Countries

**DOI:** 10.3389/fpubh.2021.652439

**Published:** 2021-06-08

**Authors:** Alexander Kwarteng, Augustina Sylverken, Daniel Antwi-Berko, Samuel Terkper Ahuno, Samuel Opoku Asiedu

**Affiliations:** ^1^Department of Biochemistry and Biotechnology, Kwame Nkrumah University of Science and Technology, Kumasi, Ghana; ^2^Kumasi Centre for Collaborative Research in Tropical Medicine, Kwame Nkrumah University of Science and Technology, Kumasi, Ghana; ^3^Department of Theoretical and Applied Biology, Kwame Nkrumah University of Science and Technology, Kumasi, Ghana; ^4^Department of Basic and Applied Biology, University of Energy and Natural Resources (UENR), Sunyani, Ghana

**Keywords:** immunology, training programs, infectious disease, research, education

## Abstract

The burden of infectious disease in developing countries is substantially higher than in developed nations. Reasons include poor health care infrastructure and deficiencies in public understanding of infectious disease mechanisms and disease prevention. While immunology education and research have an enviable role in understanding host-pathogen interactions, training programs in immunology remain fully integrated into the curricula of higher institutions, and by extension, to high schools of developing nations. Therefore, we discussed the need to make major investments in immunology research and research training into all natural sciences teaching curricula, particularly in developing countries.

## Introduction

Low and middle-income countries (LMIC), popularly referred to as developing nations, are well-known as the epicenters of many infectious diseases. Both emerging and re-emerging infections account for the high morbidity and mortality in these countries (1). These countries also bear a bigger brunt of some global infectious pathogens, such as *Mycobacterium tuberculosis*, Ebola virus, HIV, and others, including the recent novel coronavirus (SARS-CoV-2) (2, 3). In 2015 alone, over 95% of all tuberculosis-related deaths occurred in developing countries (4). An estimated 800,000 [650,000–980,000] people in Africa alone have been reported to have died from HIV/AIDS (5). *Plasmodium* infections, which disproportionally affect African countries, accounted for some 92% of all malaria-related deaths in the same year. Similarly, countries that lie within the so-called meningitis-belt continue to experience a sporadic outbreak of meningitis usually accompanied by the debilitating sequel of events even after treatment (6, 7).

Notwithstanding, the magnitude of recent COVID-19 has demonstrated that all nations (whether developed or developing) are not immune to a pandemic. However, it suggests proactive measures, including healthcare workers preparedness, establishing new research frontiers to understand host-pathogen interaction, and building sustainable health systems (8, 9). Predictive modeling by the World Health Organization estimates that ~300,000 individuals are likely to die from the novel coronavirus in Africa due to factors such as lack of access to adequate water supply, inadequate apparels (personal protective equipment), and partial or non-adherence to standard protocols (such as regular handwashing and observing appropriated physical distancing) (10, 11). Approximately 60% of urban dwellers in most African countries live in overcrowded slums, which could easily facilitate the spread of the diseases. Thus, it is not surprising that Africa has been forecasted as the potential epicenter for the novel coronavirus in the years ahead (11).

Co-infections and secondary infections have made it even more problematic in fighting some of these poverty-related infections such as HIV/TB co-infection, plasmodium/helminth infections, and among others. The number of cases for non-communicable diseases (such as cardiovascular diseases, cancer, obesity, and diabetes) is picking up in many developing countries with associated mortality rates and disability-adjusted-life-years (DALYs) (12). Although global cases of allergies and autoimmune diseases are on the rise, the incidence is less in developing countries, which may be partially explained by the hygiene hypothesis. The inverse relationship between infectious diseases and allergic/autoimmune diseases has been suggested (13), but further studies are required to explain further some of the outstanding research questions (14, 15).

Therefore, to improve health outcomes, provide a better quality of life with reduced disability-adjusted-life-years (DALYs) (16), innovative and educational policies and practices that address health challenges of developing countries are urgently needed (17).

The multidisciplinary field of immunology, which cuts across epidemiology, pathophysiology, microbiology, genetics, and environmental science, is increasingly gaining prominence in various institutions of higher learning worldwide (17). One reason for expanding immunology research is the recognition that immune dysregulation is often the underlying cause or major contributing factor to the pathogenesis of seemingly unrelated diseases such as diabetes, metabolic syndromes, or cancer. Another factor for appreciating immunology is the opportunity of genomics and using other novel high throughput approaches to advance precision medicine. This will enable us to identify risk factors or determinants of diseases, understand and monitor disease progression, identify innate protective mechanisms, develop novel diagnostics, and therapeutic interventions, track treatment strategies/options, thereby allowing more personalized and predictive health care (18).

Applications of immunology-driven precision medicine have been demonstrated in some developed countries (19) and could be exploited in resource-limited settings. Therefore, there is a clear need for translational biomedical sciences that rely on immunological skills in Africa and developing countries.

Promotion of immunology education and research can lead to the generation of a pool of researchers with specialist knowledge and skills to address community or country-specific health needs given the rampant global pandemics such as the current COVID-19 and Ebola diseases. Immunology education and research can, as well, aid in the development of tools and equipment much more suited for developing countries.

Interestingly, there are many professions aside from faculty and research positions in higher learning institutions that rely on the skills and expertise of immunologists. While the current state of immunology research and education in developing countries has not been comprehensively evaluated, there are few academic and research institutions, for instance, in sub-Saharan Africa where immunology programs and research are conducted. In addition, in developing countries, is not only the number of immunology faculty, teaching vs. research laboratories, and graduate programs a concern, but also resources available to effectively run immunology-based programs need much attention in order to achieve the targeted goals.

A number of grant agencies and biomedical research and development organizations require the skills of the immunologist to choose research priority areas, assess the suitability of grant applications, and provide high-level feedback on the feasibility and impact of supposed research as progress reports. Various governmental agencies also need the services of immunologists to inform policy regarding immunization, effective response to epidemics/pandemics, and the regulation and standardization of environmental factors that impact immune response and behavior of societies in general (20). Although vaccines are an important part of the family and public health, not many people embrace them due to limited knowledge about their roles in preventable diseases. For example, there was a public uproar and campaign against malaria and Ebola vaccine trials in Ghana, which could be attributed to fear and limited knowledge. Obviously, there is a need for more community-focused interaction and engagement to increase public understanding of the safety and importance of vaccine use to ensure maximum participation in vaccine programs.

Immunologists could also play key roles in community engagement, clearly articulate specialized knowledge to lay people in the community on vaccination, the immune response to diseases, counseling parents and guidance on inborn errors of immunity, and management of allergies autoimmune diseases. Such engagements will also promote more research on identifying and developing an effective vaccine against other diseases that disproportionately affects vulnerable groups such as children, the elderly, and pregnant women in most LMICs. The immunologist's roles also extend to administrative, sales, supervisory roles in biotechnology and private industries, particularly pharmaceutical companies, although such industries are limited in LMICs.

In developing nations, research skills such as evaluation of clinical data and developing predictive models are needed in addition to skills in immunological techniques such as Next-Gen Sequencing, flow cytometry, mass spectrometry, and Enzyme-Linked Immunosorbent Assays (ELISA).

Therefore, there is a need for immunology programs with robust research components in higher institutions in developing countries that seek to address the health needs of LMICs specifically. This could also have an indirect effect on improved detection, diagnosis, and treatment of diseases that rely on developing countries' immune techniques. Therefore, we propose innovative strategies of increasing immunology education at basic, middle, and higher institutions of learning and strengthening immunology-based research in developing nations.

## Toward a Holistic Immunology Education

There is increasing evidence that immune behavior in health and diseases is affected by socioeconomic status (SES), genetic, epigenetic, molecular, cellular, and environmental modulatory factors (21). A better understanding of these multifactorial linkages will be almost impossible without exploiting all aspects of immunology; clinical, experimental, and computational immunology strategies (22). The need to develop novel animal models that mimic complex diseases is critical for creating new mathematical and computational models to predict immune responses (22, 23). This alone may not answer the challenges in immunology research but will have to go along with the development and availability of technological innovations with minimal sample preparation steps, shorter runtimes, and high-quality results (higher sensitivity, specificity, and accuracy). A holistic immunology education that captures clinical, experimental, and computational immunology concepts, particularly in developing countries, must be pursued to obtain quality results.

Given the interdisciplinary nature of immunology, trainees must acquire skills in genomics, proteomics, and other omics disciplines such as pathway biology, among others. In addition, knowledge of the software that accompanies major immunoassays as well as statistical computing software such as R and Python maybe acquired.

Currently, immunology courses are mostly pursued at the postgraduate levels even though a few undergraduate programs such as biological sciences teach some related immunology courses. This could be explained by several factors such as the availability of faculty, research facilities, cost of reagents, students' interest, job prospects, and other things. However, recent calls have been made to introduce immunology courses at the undergraduate levels (24). Similarly, basic immunological concepts can be introduced in high schools to appreciate the roles and relevance of immunology in disease and health. In Ghana, for instance, the concept of immunology is introduced on average at age 17 years compared to the US, where the average age is around 15 years; this makes a compelling case that students in Ghana should learn the concept of immune response earlier and that the link to epidemiology aspects should be strengthened.

Given that the immune system contributes to all aspects of human health and disease, immunology and its connections with all other physiology should indeed be taught at high school and undergraduate levels. In developing countries where the infectious disease burden is greater, this is even more important. Learning materials such as audio-visuals, multimedia, and teaching aids with better-resourced laboratories to connect the complex themes and keywords to the learner could help promote immunology education in the country.

The internet is now replete with free immunology courses, thereby cutting down the cost of education, improving access, and promoting equal opportunity to quality educational resources. It also offers the opportunity to spark students' interest in learning immunology and keep them motivated throughout their education and career. The sustained interest could also lead to scientific discoveries in health and medicine. However, it must be indicated that the onset of immunology programs at the undergraduate levels and beyond at higher institutions in developing countries will require extensive preparation and dedicated resources such as facilities, faculty, equipment, mentors, and other stakeholders.

## Effective Mentorship; Standing on the Shoulders of Giants

To fully explore the prospects of immunology in developing countries, the role of mentors cannot be underestimated. Mentors play key roles in nurturing tomorrow's scientific leaders. Effective mentorship is, therefore, a contributing factor to good research and higher academic outcomes. Matching trainees with appropriate mentors, either locally or internationally, could prepare and nurture young investigators. However, this is already a challenge due to the limited number of immunologists in developing countries. Conversely, many institutions have recognized the challenge, leading to virtual and physical mentorship programs in developing countries, such as the AuthorAid mentorship program.

The mentorship program hosted by AuthorAid, Beyond Sciences Initiatives (BSI), and other biomedical and immunology societies are innovative practices to connect established scientific leaders with upcoming ones. There is an increasing trend in the number of immunology societies across developing countries moderated by the International Union of Immunological Societies (IUIS) and Federation of African Immunological Societies (FAIS). This signifies the importance of immunology in advancing basic research and developments, particularly in the area of infectious diseases. However, these programs by themselves are not enough, therefore, warranting the need for an extra effort by various scientific societies, academic, and research institutions to liaise with immunology and vaccinology-based industry players, and other established academics researchers to mentor the next generation of immunologists.

Mentorship can be in research collaborations and training where mentees can spend an allocated period in research laboratory/groups of mentors. In addition, such mentorship programs could be made to include women and minority groups that are severely underrepresented in immunology education and research. As is the dearth of immunology mentors in developing countries, there is a need for more international collaboration in research and research training.

## The Role of Governments

Effective training of immunologists will demand huge capital and resource investment, given the high cost of establishing and maintaining immunology core facilities, running experiments, demonstrations, and practical sessions. Although immunology experiments could be less predictable with a little direct effect on patients in the short-term, the long-term benefits of supporting immunology research and education are just enormous. It is a genuine concern that developing countries have other urgent initiatives to support limited resources; however, given the benefits of immunology education and research, it will be prudent for countries to dedicate adequate funds and maintain commitments toward immunology education and research, thereby addressing the deficit of immunologists in developing countries. The high cost of immunology research in developing countries could be supplemented with sustainable international or regional collaborations.

## Role of National, Regional, and Global Agencies/Associations

Non-governmental organizations can play major roles in the promotion of immunology research and education in developing countries. Agencies such as the African Academy of Sciences, various national immunology societies, and the International Union of Immunology Societies, Volkswagen (VW) foundation do facilitate knowledge sharing through workshops, conferences, summer schools to teach and train budding immunologists with new techniques and to even inform policies and practices. Alternatively, funding agencies and immunology societies could support online training programs for students and early-career scientists.

## Conclusion

This paper highlights the prospects of immunology as an important discipline in developing countries in the wake of emerging and re-emerging infectious diseases, such as Ebola and SARS-COV2. Immune dysregulation underlies and/or contributes to almost every human disease—hence immunology should be taught as an integral part of introductory studies of human biology/physiology, starting at the basic and middle level of education in low-and middle-income countries. While governments of developing nations can be called to assure infrastructure that allows for research, international collaborations should focus on research and sustainable partnerships that ensure research training, including mentorship and capacity building.

## Author Contributions

All authors listed have made a substantial, direct and intellectual contribution to the work, and approved it for publication.

## Conflict of Interest

The authors declare that the research was conducted in the absence of any commercial or financial relationships that could be construed as a potential conflict of interest.
